# Occurrence of *Rickettsia felis *in dog and cat fleas (*Ctenocephalides felis*) from Italy

**DOI:** 10.1186/1756-3305-2-S1-S8

**Published:** 2009-04-20

**Authors:** Gioia Capelli, Fabrizio Montarsi, Elena Porcellato, Giulia Maioli, Carmelo Furnari, Laura Rinaldi, Gaetano Oliva, Domenico Otranto

**Affiliations:** 1 Istituto Zooprofilattico Sperimentale delle Venezie, Legnaro, Padova, Italy; 2 Istituto Zooprofilattico della Lombardia e dell'Emilia Romagna, Reggio Emilia, Italy; 3 ULSS 20, Verona, Italy; 4 Department of Pathology and Animal Health, Faculty of Veterinary Medicine, University of Naples "Federico II", Italy; 5 Department of Veterinary Clinical Science, Faculty of Veterinary Medicine, University of Naples "Federico II", Italy; 6 Department of Veterinary Public Health and Veterinary Sciences, Faculty of Veterinary Medicine, University of Bari, Italy

## Abstract

*Rickettsia felis *is an obligate intracellular bacterium belonging to the spotted fever group, suspected to cause a murine typhus-like illness in humans, with a cosmopolitan distribution. This study was designed to estimate presence and occurrence of this pathogen in fleas collected from dogs and cats in different areas of Italy. Two species of fleas were identified, *Ctenocephalides felis *(80.3%) and *Ctenocephalides canis *(19.7%).

Overall, 320 fleas (257 *C. felis *and 63 *C. canis*) collected from 117 animals (73 dogs and 44 cats) were tested. Thirty-eight (11.9%) *C. felis *fleas, 13 from cats (17.6%) and 25 from dogs (10.2%) were positive for *R. felis*. No *C. canis *was positive. Fleas from cats showed a tendency to be more positive than fleas from dogs. Prevalence of *R. felis *among areas and within provinces of the same area was extremely variable, ranging from 0 to 35.3%. Overall, prevalence in north-eastern Italy (23.2%) was significantly higher than in south-western Italy (7.1%). This study confirmed the occurrence of *R. felis *in cat and dog fleas (*C. felis*) from Italy, similar to other European countries. The results also suggest that *R. felis *should be considered in the human differential diagnosis of any spotted-like fever in Italy, especially if the patient is known to have been exposed to flea bites.

## Findings

*Rickettsia felis *is an obligate intracellular bacterium belonging to the spotted fever group, suspected to cause a murine typhus-like illness in humans [1-3]. Following its first detection [4] in midgut cells of the cat flea *Ctenocephalides felis*, this organism has been later described as a new species [5].

Biological and genomic investigations have shown that *R. felis *displays unique features compared with other rickettsieae, in particular in its genomic [6,7] and laboratory cultivation [8]. Molecular epidemiological investigations allowed detection of *R. felis *in fleas from different geographical areas of the world, further supporting the cosmopolitan distribution of this pathogen [1].

In Italy, *R. felis *has been detected in cat and dog fleas limitedly to an area of north-eastern Italy [9]. The aim of the present study was to obtain further information on the occurrence and distribution of *R. felis *in fleas from different Italian regions, where the presence of this agent is still unknown.

From March 2008 to March 2009, fleas were collected from owned animals, colony cats, stray and kennelled dogs of four provinces of Veneto region (north-eastern Italy), two provinces of Campania region (south-western Italy) and of Bari province (Apulia region, south-eastern Italy). All fleas, preserved in isopropanol, were morphologically identified and sexed by using light microscopy and following an identification key [10]. A range of 1–3 fleas per animal host were randomly chosen for molecular analyses.

DNA extraction was performed using a commercial kit (DNeasy^® ^Blood&Tissue Qiagen kit). A 401-bp fragment of the rickettsial gltA (citrate synthase) gene was PCR-amplified using CS-78 (forward) and CS-323 (reverse) primers [11].

Briefly, PCRs (50 μl) were performed in an Applied Biosystems Thermocycler (Gene Amp PCR System 9700), adding 5 μl of the DNA template to 31.7 μl of the molecular-grade water, 5 μl of buffer 10×, 3 μl of MgCl_2 _25 mM, 1 μl of dNTPs 10 mM, 2 μl of each primer (CS-78 and CS-323) 10 μM and 3 μl of AmpliTaq Gold 5 U. PCR cycling conditions were as follows: 1 initial cycle at 94°C for 10', 35 cycles of 15" at 95°C, 30" at 55°C, and 30" at 72°C, and 1 final step at 72°C for 7'.

The complete coding sequences were generated using the Big Dye Terminator v3.1 cycle sequencing kit (Applied Biosystem, Foster City, CA, USA). The products of the sequencing reactions were purified using PERFORMA DTR Ultra 96-Well kit (Edge BioSystems, Gaithersburg, MD, USA) and sequenced in a 16-capillary ABI PRISM 3130×l Genetic Analyzer (Applied Biosystem, Foster City, CA, USA). Sequences obtained were compared with those of characterised rickettsieae in Genbank by using BLAST analysis .

Prevalence differences in relation to host species (cats and dogs), provenance and flea sex were tested by χ^2 ^test or Fisher's Exact test using the SPSS statistical package (SPSS Inc., USA) for Windows, version 15.0.

Two species of fleas were identified, *C. felis *(80.3%) and *Ctenocephalides canis *(19.7%). Cats were infested only by *C. felis *and dogs by both species, with *C. felis *again as the predominant flea (75%).

**Figure 1:**
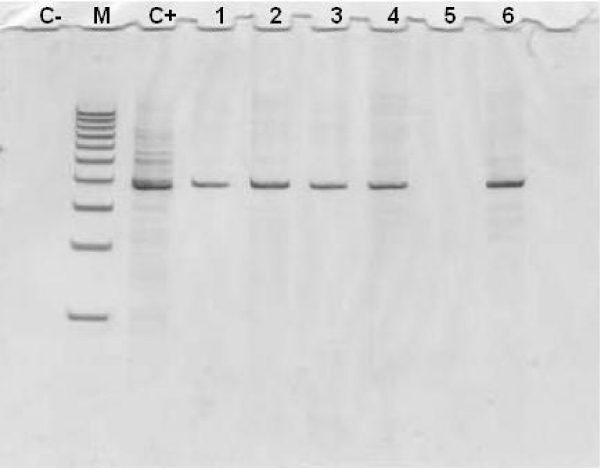
**Image of an acrylamide gel 7% showing PCR amplification of the 401-bp fragment (*gltA *rickettsial gene)**. C+ = positive control; C- = negative control (distilled water); 1–6 = flea samples (1,2,3,4,6 positives); M = molecular weight (100 bp PCR Molecular Ruler, BioRad, Laboratories Inc., Hercules, CA, USA).

The results of the molecular study are summarised in Table 1. Overall, 320 fleas (257 *C. felis *and 63 *C. canis*) collected from 117 animals (73 dogs and 44 cats) were tested. Thirty-eight (11.9%) *C. felis *fleas, 13 from cats (17.6%) and 25 from dogs (10.2%) were positive for *R. felis *yielding PCR bands of the expected size (Figure 1). No *C. canis *was positive. Sequenced amplicons displayed an overall 99% similarity to *R. felis *sequence available in the Genbank database (*Rickettsia felis *URRWXCal2; accession number CP000053.1).

**Table 1:** Number of fleas tested and positive for *Rickettsia felis *and number of animals on which fleas were collected from different geographical areas of Italy.

Species	Province (area of Italy)	Examined fleas/animals^a^	Positive fleas/animals	*R. felis *prevalence in fleas, %
Cat	Belluno (ne)	1/1	0	-
	Padova (ne)	9/9	2/2	22.2
	Venezia (ne)	39/13	5/3	12.8
	Verona (ne)	17/15	6/6	35.3
	Napoli (sw)	8/6	0	0.0
	Total fleas/cats	74/44	13/11	17.6
				
Dog	Belluno (ne)	1/1	1/1	-
	Padova (ne)	34/9	10/2	29.4
	Venezia (ne)	6/3	1/1	16.7
	Verona (ne)	5/2	1/1	20.0
	Napoli (sw)	97^b^/29	7/5	7.2
	Caserta (sw)	62^c^/8	5/4	8.0
	Avellino (sw)	2/1	0	-
	Bari (se)	39/20	0	0.0
	Total fleas/dogs	246/73	25/14	10.2
				
	Total	320/117	38/25	11.9

^a ^fleas/animals = number of fleas examined and collected from a number of animals.

^b ^Including 62 *Ctenocephalides canis *fleas.

^c ^Including one *Ctenocephalides canis *flea.

se, south-eastern Italy; ne, north-eastern Italy; sw, south-western Italy.

Specifically, positive fleas were found from both dogs and cats in all four provinces of north-eastern Italy, from dogs in two provinces of south-western Italy (Naples and Caserta), whereas all fleas from Bari province (southeastern) were negative (Table 1).

Fleas from cats showed a tendency (Fisher's exact test; p = 0.068) to be more positive (17.6%) than fleas from dogs (10.2%). Male and female fleas showed a similar rate of infection (11.6% and 8.9%, respectively). Prevalence of *R. felis *among areas and within provinces of the same area was extremely variable, ranging from 0 to 35.3% (Table 1). In general and excluding fleas from Bari province (a single site of sampling) prevalence in north-eastern Italy (23.2%) was significantly higher than in south-western Italy (7.1%) (χ^2 ^test = 14.956; p < 0.01).

Our results indicate that *R. felis *is present in *C. felis *fleas from several geographical locations of Italy. The occurrence of *R. felis*-positive fleas from dogs and cats in north-eastern and south-western Italy is similar to data available in other European countries, i.e. Germany [12], France [13] and Spain [14].

In this study, *R. felis *was detected for the first time in south-western Italy, whilst the negative results from south-eastern Italy require further investigations, being fleas collected only in dogs housed in a sole municipal kennel. The higher prevalence of *R. felis*-infected fleas in north-eastern Italy compared with south-western areas is currently difficult to explain, a sampling bias can not be excluded and only further and more balanced sampling will confirm this trend.

In our study, *C. felis *was the only flea species infected as reported by several other authors, nevertheless *C. canis *was previously found as a *R. felis *carrier in other investigations, along with others flea species, ticks and mites (reviewed in [2]). A possible explanation for the negativity of *C. canis *in our study could be that this flea was found only in dogs not showing mixed infection with *C. felis *and presumably not infected with *R. felis*. This hypothesis would explain also why *C. felis *fleas were found more infected when collected on cats rather than on dogs. However, the mechanism of *R. felis *transmission to vertebrates and to uninfected fleas in nature is still unknown, even though there is experimental evidence indicating that *R. felis *is maintained in cat fleas primarily by transovarian and transstadial transmission [15].

Besides vertical transmission, the first identification of the bacterium in salivary glands of *C. felis *[16], along with the evidence of seroconvertion and *R. felis *DNA detection in blood from cats exposed to infected fleas [17], strongly supports the potential for horizontal transmission among vectors and to vertebrate hosts. Among the other possible mammal hosts of *C. felis*, several studies [3] have shown that opossums seroconvert, and are usually heavily infested with the infected cat fleas. Thus, opossums could indirectly serve as a bridge for the transmission of *R. felis *to vertebrates.

Furthermore, a study conducted in Germany [12] in fleas collected from cats and dogs, provided evidence that *Archeopsylla erinacei *(the hedgehog flea) carried *R. felis *in all the specimens tested, compared to a low positivity found in *C. felis *(9%). The data above suggest that *A. erinacei *could have played a role in transmitting *R. felis *to humans in Germany and indicate the hedgehog as another candidate to be a potential reservoir of the infection.

Opossums and other vertebrate hosts may play a role in rickettsial horizontal transmission to other ectoparasites, and this may account for the occasional reports of *R. felis *infection in other flea species as well as ticks.

Clinical symptoms have not been reported in any animal carrying positive fleas. The current knowledge suggests that the only role of mammals infected by *R. felis *is likely to amplify the cycle by fleas feeding on their *R. felis*-infected blood. This hypothesis is supported by the fact that even if vertical transmission of *R. felis *persists in *C. felis *for at least 12 generations not feeding in a *R. felis*-infected host, over successive generations prevalence shows a natural decrease [18].

Actually, *R. felis *has been associated with diseased hosts only in humans. Infection by *R. felis *in humans has been reported in USA, Mexico, Brazil, France and Spain [1,2] both by serological and molecular evidence, but unfortunately the pathogen has never been successfully isolated from humans. The lack of a human isolate of *R. felis *does not permit the definition of this organism as a confirmed human pathogen.

To our knowledge no human cases of rickettsiosis due to *R. felis *have been diagnosed or suspected in Italy. However, clinical symptoms for *R. felis *infections are similar to those of other rickettsial diseases, which make difficult an aetiological diagnosis based only on the clinical presentations. Conversely, spotted fever rickettsiosis due to *R. conorii *in Italy is endemic in southern regions while some sporadic case of murine typhus due to *R. typhi *has been reported and a few cases were serologically attributed to *R. helvetica *[19]. Recently, *R. slovaca *has been identified in ticks removed from humans showing a tick-borne lymphadenopathy in the Tuscany region [20].

This study confirmed the presence and diffusion of *R. felis *in cat and dog fleas (*C. felis*) from Italy, similar to other European countries [21]. The results also suggest that *R. felis *should be considered in the human differential diagnosis of any spotted-like fevers in Italy, especially if the patient is known to have been exposed to flea bites. Nonetheless, the role of mammals, mainly dogs and cats, in the epidemiology of this flea-borne infection needs further confirmatory evidence. To unravel the close relationship between *R. felis *and *C. felis *more studies are needed in rickettsial infection dynamics in the flea vector, which likely will further clarify the ecology and epidemiology of *R. felis *transmission in nature.

## Competing interests

The authors declare that they have no competing interests.

## Authors' contributions

GC, FM and GM conceived and designed the experiments; EP and GM carried out the molecular genetic analyses; GM, CF, LR, GO and DO have made substantial contributions to acquisition of fleas and related data; GC and FM analysed and interpreted the data and wrote the paper; GO, LR and especially DO revised the article critically for important intellectual content.
